# Wild-type ALK and activating ALK-R1275Q and ALK-F1174L mutations upregulate Myc and initiate tumor formation in murine neural crest progenitor cells

**DOI:** 10.18632/oncotarget.2036

**Published:** 2014-05-27

**Authors:** Gisèle Montavon, Nicolas Jauquier, Aurélie Coulon, Michel Peuchmaur, Marjorie Flahaut, Katia Balmas Bourloud, Pu Yan, Olivier Delattre, Lukas Sommer, Jean-Marc Joseph, Isabelle Janoueix-Lerosey, Nicole Gross, Annick Mühlethaler-Mottet

**Affiliations:** ^1^ Pediatric Department, HFR Hospital Fribourg, Villars-sur-Glâne, Switzerland; ^2^ Pediatric Surgery, Pediatric Department, University Hospital CHUV, Lausanne, Switzerland; ^3^ Pediatric Oncology Research, Pediatric Department, University Hospital CHUV, Lausanne, Switzerland; ^4^ Service d'anatomie pathologique, Hôpital Robert Debré, Paris, France; ^5^ Pediatric Pathology, Pathology Department, University Hospital CHUV, Lausanne, Switzerland; ^6^ INSERM U830, Institut Curie, Centre de Recherche, Paris, France; ^7^ Cell and Developmental Biology, Institute of Anatomy, University of Zürich, Zürich, Switzerland

**Keywords:** ALK, neuroblastoma, Myc, tumorigenesis, differentiation

## Abstract

The anaplastic lymphoma kinase (*ALK*) gene is overexpressed, mutated or amplified in most neuroblastoma (NB), a pediatric neural crest-derived embryonal tumor. The two most frequent mutations, ALK-F1174L and ALK-R1275Q, contribute to NB tumorigenesis in mouse models, and cooperate with MYCN in the oncogenic process. However, the precise role of activating ALK mutations or ALK-wt overexpression in NB tumor initiation needs further clarification.

Human ALK-wt, ALK-F1174L, or ALK-R1275Q were stably expressed in murine neural crest progenitor cells (NCPC), MONC-1 or JoMa1, immortalized with v-Myc or Tamoxifen-inducible Myc-ER^T^, respectively. While orthotopic implantations of MONC-1 parental cells in nude mice generated various tumor types, such as NB, osteo/chondrosarcoma, and undifferentiated tumors, due to v-Myc oncogenic activity, MONC-1-ALK-F1174L cells only produced undifferentiated tumors. Furthermore, our data represent the first demonstration of ALK-wt transforming capacity, as ALK-wt expression in JoMa1 cells, likewise ALK-F1174L, or ALK-R1275Q, in absence of exogenous Myc-ER^T^ activity, was sufficient to induce the formation of aggressive and undifferentiated neural crest cell-derived tumors, but not to drive NB development. Interestingly, JoMa1-ALK tumors and their derived cell lines upregulated Myc endogenous expression, resulting from ALK activation, and both ALK and Myc activities were necessary to confer tumorigenic properties on tumor-derived JoMa1 cells i*n vitro*.

## INTRODUCTION

Neuroblastoma (NB) is a heterogeneous childhood malignancy from embryonic origin arising from neural crest progenitor cells (NCPC) of the sympathoadrenal lineage [1]. Neural crest (NC) is a transient highly migratory population of neuroectodermal pluripotent stem cells in vertebrate embryo. NC cells from the trunk migrate and differentiate to give rise to glia, neurons of the dorsal root ganglia, Schwann cells, melanocytes, and adrenal medulla [2-4]. NB is believed to originate from a subset of these migratory NC derivatives committed to the sympathoadrenal lineage, which differentiate into adrenal chromaffin cells or sympathetic ganglia [1, 4].

The anaplastic lymphoma kinase (ALK) gene belongs to the insulin receptor superfamily of receptor tyrosine kinase (RTK). ALK has been recently extensively studied as a candidate in the development of new targeted treatments for progressive and resistant NB. ALK is expressed in the developing central and peripheral nervous system during embryogenesis [5], and in the developing sympathoadrenal lineage of the NC, where its signaling may regulate the balance between cell proliferation and differentiation [1, 6, 7]. ALK physiological role in the normal development of the nervous system is not yet fully understood, but ALK having a role in neurogenesis control was demonstrated in *Drosophila*, zebrafish, and chicken [6, 8, 9].

ALK mutations were described in ~10% of NB cases, copy number gain in ~15%, and ALK amplification in 2-4% of NB [1, 10-16]. Furthermore, overexpression of ALK-wt or mutated receptors was detected in the majority of NB, and was shown to be associated with poor clinical outcome [17, 18]. The two most frequent mutations F1174L (only present in sporadic NB) and R1275Q (in sporadic and familial NB) correspond to 34.7% and 49% of mutated ALK, respectively [10, 15, 19]. Both mutations lead to ALK constitutive activation by autophosphorylation, although ALK-F1174L displays a higher degree of phosphorylation, and an increased tumorigenic potential compared to other ALK mutations [10, 13, 20-22, 26]. Both activating mutations, ALK-F1174L and ALK-R1275Q, were also shown to contribute to NB tumorigenesis in transgenic and knock-in (KI) animal models, as well as to cooperate with MYCN in the oncogenic process [23-26]. In patient, although association of ALK-F1174 hotspot mutations with M*YCN* amplification is still under debate [10, 11], co-occurrence of both genetic alterations lead to very poor outcome compared to single defect.

To date, the role of activating ALK mutations or ALK-wt overexpression in NB tumor initiation and progression remains unclear. Indeed, although germline activating ALK mutations occur in 80% of familial NB, they display incomplete penetrance suggesting that additional genetic alterations may be required for NB initiation [15, 16, 27]. Moreover, ALK-F1174L expression, in mouse and zebrafish transgenic NB models, as well as in KI model, was not sufficient to initiate NB tumor formation in absence of MYCN co-expression or additional genetic alterations syntenic to that commonly found in human NB [23-25, 26]. This suggests that ALK-F1174L, similarly to germline ALK mutations, required secondary hits to drive NB formation [23].

In this study, we investigated the ability of ALK-wt, and the most common activating mutations, ALK-F1174L and ALK-R1275Q, to initiate tumor formation in NCPC, and we compared their *in vivo* oncogenic potential. In this aim, two murine NCPC models were selected, the MONC-1 cell line immortalized with v-Myc [28], and the JoMa1 cell line expressing a Tamoxifen-inducible Myc-ER^T^ [29], allowing evaluation of ALK-wt and variant functions in presence or absence of exogenous Myc activity. Stable expression of ALK-wt or gain-of-function mutants in NCPC were sufficient to induce formation of highly aggressive and undifferentiated tumors, but not to drive NB tumor progression. Moreover, Myc endogenous expression was strongly upregulated in orthotopic JoMa1-ALK tumors or their derived cell lines as a result of ALK activation, and both ALK and Myc activities were required to maintain *in vitro* tumorigenic capacities of tumor-derived cell lines. These data strongly support a role for ALK-wt, in addition to ALK-F1174L and ALK-R1275Q, to confer *in vitro* and *in vivo* tumorigenic properties on NCPC.

## RESULTS

### ALK-F1174L expression in murine NCPC MONC-1 impairs differentiation of NC cell-derived tumors

**Figure 1:**
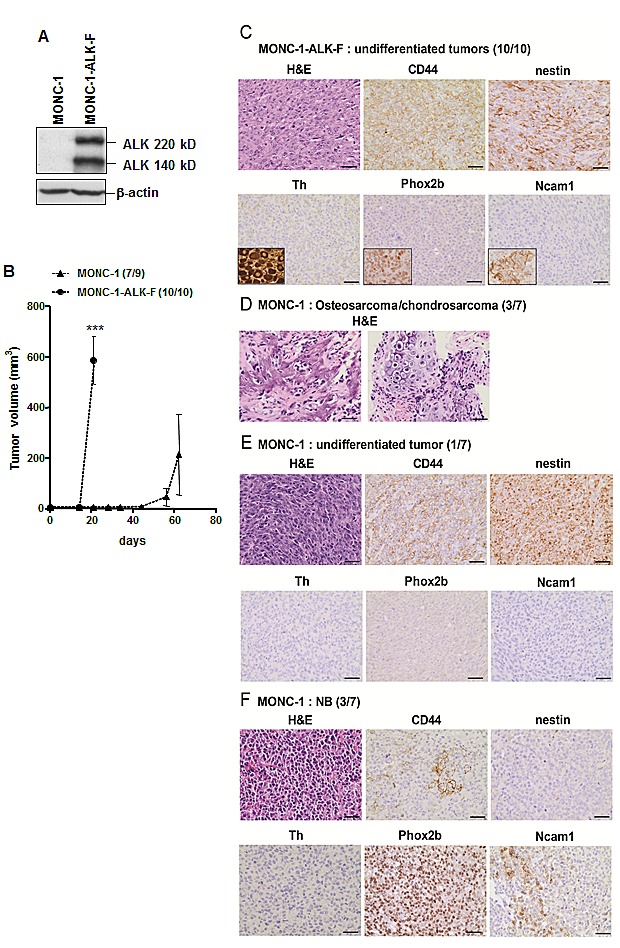
ALK-F1174L impairs differentiation of MONC-1-derived tumors A. Whole cell extract of MONC-1 parental cells and MONC-1-ALK-F1174L transduced cells were analyzed by immunoblotting for the presence of human ALK. β-actin was used as loading control. B. Tumor take (number of tumor-bearing mice /total nude mice) and growth (mean tumor volumes ± SEM) of MONC-1 and MONC-1-ALK-F cells orthotopically implanted and measured by echography (unpaired t test with Welch's correction, ***=p<0.0001). C. H&E and IHC analyses for various markers are shown for one representative tumor derived from MONC-1-ALK-F1174L cells (magnification 40x, scale = 20 μm). Positive controls for Th (ganglion), Phox2b (NB), and for Ncam1 (adrenal gland) are shown in small inserts. D. H&E analysis of one representative osteosarcoma tumor with chondrodarcoma componant derived from MONC-1 cells (left: osteosarcoma, right: chondrosarcoma). E. The undifferentiated tumor derived from MONC-1 cells is shown. F. One representative NB tumor derived from MONC-1 cells is shown.

To investigate the oncogenic potential of ALK-F1174L mutation in NCPC, human ALK-F1174L was overexpressed in the murine NCP cell line, MONC-1, previously immortalized by stable v-Myc expression [28] (Figure 1A). Transduced MONC-1 cells conserved their NCPC phenotype, as the NC stem cell (NCSC) markers, except Sox10, were still expressed, while glial or neuronal differentiation markers were not detected (Supplementary Figure 1A). The tumorigenic potential of MONC-1-ALK-F1174L or parental MONC-1 cells was analyzed *in vivo* by orthotopic implantation into nude mice adrenal glands (AG). Interestingly, mice implanted with MONC-1-ALK-F1174L cells developed highly aggressive tumors in all mice (10/10, 100%) within three weeks, while mice engrafted with parental MONC-1 cells developed tumors in AG with a significantly longer latency (7/9, 78%)(Figure 1B). MONC-1-ALK-F1174L-derived tumors strongly expressed human ALK mRNA and protein as expected, but not murine Alk (Supplementary Figure 1B). Thus, ALK-F1174L strongly accelerated MONC-1 cell-derived tumor growth.

All mice implanted with MONC-1-ALK-F1174L cells developed highly malignant undifferentiated tumors, as they strongly expressed the mesechymal/stem marker CD44 and the neural stem/progenitor cell marker nestin, but did not stain for the neuronal marker Ncam1, the adrenergic differentiation marker tyrosine hydroxylase (Th), and the sympathoadrenal marker Phox2b, recently demonstrated as a highly specific marker of undifferentiated NB [30] (Figure 1C). In contrast, MONC-1 cells gave rise to various tumor types, as 3/7 mice developed osteosarcoma with chondrosarcoma components (Figure 1D), 1/7 mouse developed a highly malignant Phox2b^−^/nestin^+^ undifferentiated tumor (Figure 1E), and 3/7 mice developed Phox2b^+^/Th^−^/nestin^−^ undifferentiated NB (Figure 1F). The three MONC-1-derived NB tumors displayed features of unfavorable NB as seen in patients, such as stroma poor and high MKI (data not shown). These NB tumors expressed reduced levels of CD44, but increased levels of Ncam1, compared to undifferentiated tumors derived either from MONC-1 or MONC-1-ALK-F1174L cells (Figure 1C,,). Altogether, these results suggest that v-Myc, constitutively expressed in MONC-1 cells, enables the formation of diverse differentiated tumors corresponding to various NCPC derivates. Moreover, ALK-F1174L expression is highly tumorigenic in MONC-1 cells, and seems to impair NCPC differentiation, as MONC-1-ALK-F1174L cells only produced highly undifferentiated NC cell-derived tumors.

### JoMa1 cells expressing ALK-wt, ALK-F1174L, or ALK-R1275Q are tumorigenic *in vivo*

**Figure 2:**
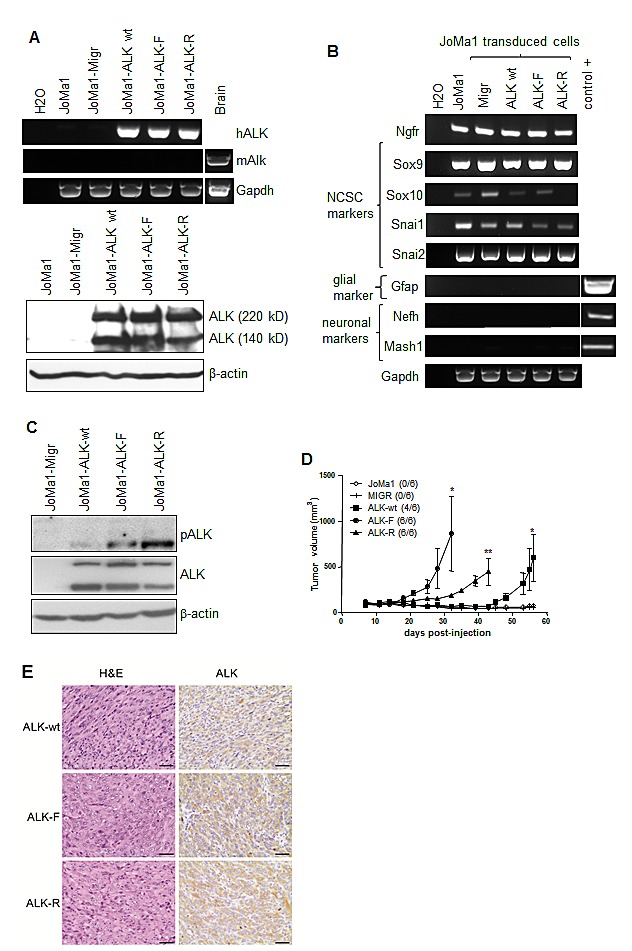
ALK-wt-, ALK-F1174L-, and ALK-R1275Q-expressing JoMa1 cells are tumorigenic in subcutaneous *in vivo* model A. Analyses of ALK mRNA and protein expressions in parental JoMa1 cells and in JoMa1 cells transduced with the empty vector (Migr) or vector encoding for ALF-wt, ALK-F1174L or ALK-R1275Q. Murine GAPDH and β-actin were used as control for RT-PCR (top) or immunoblotting (bottom), respectively. B. Expression levels of various NCSC and differentiation markers analyzed by RT-PCR in total RNA from JoMa1 parental and transduced cells. C. Immunoblotting analysis of ALK phosphorylation in transduced JoMa1 cells. β-actin was used as loading control. D. Tumor take (number of tumors/total implanted mouse flanks) and growth (mean tumor volumes ± SEM) of JoMa1 parental and transduced cells implanted subcutaneously in both flanks of nude mice are represented (one way Anova *=p<0.05, **=p<0.005) E. H&E, and IHC-staining for ALK of one representative subcutaneous tumor derived from JoMAa1-ALK-wt, -ALK-F1174L, and -ALK-R1275Q cells (magnification 40x, scale = 20 μm).

We next addressed the oncogenic potential of ALK-wt and ALK-R1275Q mutation, as compared to ALK-F1174L variant, in NCPC in absence of v-Myc oncogene expression. For this purpose we used the murine NCP cell line, JoMa1, expressing Tamoxifen (4-OHT)-inducible Myc-ER^T^ [29]. Human ALK-wt, ALK-F1174L, and ALK-R1275Q variants were stably overexpressed in JoMa1 cells, while endogenous murine Alk was undetectable in these cells (Figure 2A). NCPC phenotype of transduced JoMa1 cells was not affected by retroviral infections and ALK expression. Indeed, except for Sox10 expression which was undetectable in JoMa1-ALK-R1275Q cells, transduced cells still expressed the NCSC markers Ngfr, Sox9, Snai1, and Snai2, while glial, or neuronal differentiation markers were not detected (Figure 2B). Sustained ALK activation was detected by immunoblotting in JoMa1-ALK expressing cells (Figure 2C).

The *in vivo* tumorigenic potential of JoMa1-ALK-F1174L, -ALK-R1275Q, and -ALK-wt cells was then assessed after subcutaneous engraftment in absence of 4-OHT, thus without Myc-ER^T^ activation. Interestingly, JoMa1-ALK-wt cells were able to drive tumor formation, likewise JoMa1-ALK-R1275Q, and JoMa1-ALK-F1174L cells, while JoMa1-Migr and JoMa1 control cells did not induce tumor development (Figure 2D). ALK-F1174L conferred an enhanced tumorigenic potential to JoMa1 cells when compared to ALK-R1275Q and ALK-wt (Figure 2D). Human exogenous ALK expression in JoMa1-derived subcutaneous tumors was confirmed by IHC and RT-PCR analyses, while murine Alk mRNA was undetected (Figure 2E and Supplementary Figure 2). Thus, in absence of any exogenous Myc activation, ALK-wt or ALK activating mutations displayed transforming capacities in NCPC.

### JoMa1-ALK-wt, -ALK-F1174L, and -ALK-R1275Q cells generate undifferentiated NC cell-derived tumors *in vivo* upregulating NCSC or SC associated markers

**Figure 3:**
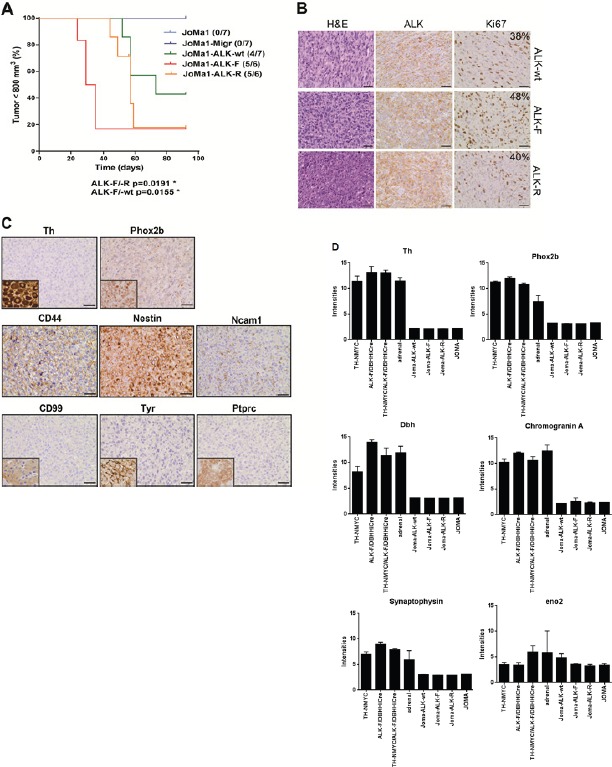
*In vivo* orthotopic implantations of ALK-wt, ALK-F1174L, and ALK-R1275Q cells generate undifferentiated NC cell-derived tumors A. Kaplan-Meier survival curves of orthotopically engrafted mice. Orthotopic tumor growth was followed by echography and mice were sacrificed once tumor on left AG reached approximately 800 mm^3^: JoMa1-ALK-F1174L, JoMa1-ALK-R1275Q, and JoMa1-ALK-wt group of mice were sacrificed from 24 to 35, 44 to 59, and 52 to 73 days post-injection, respectively (Gehan-Breslow-Wilcoxon test). All remaining mice were sacrificed 3 months post-injection. B. H&E and IHC staining for ALK and Ki67 of one representative orthotopic tumor of JoMa1-ALK-wt, -ALK-F1174L, and -ALK-R1275Q groups (magnification 40x, scale = 20 μm). C. IHC of various markers in one orthotopic JoMa1-ALK-F1174L tumor representative of all subcutaneous and orthotopic tumors (magnification 40x, scale = 20 μm). Positive controls for Th (ganglion), Phox2b (NB), CD99 (islet of Langerhans), Tyr (melanoma), and Ptprc (CD45) (spleen) are shown in small inserts. D. Bare graph showing expression levels of different genes in JoMa1-ALK tumors, NB murine transgenic model, JoMa1 cell lines, and adrenal glands.

Orthotopic implantations were then performed in order to faithfully reproduce NB tumor microenvironment. As observed in subcutaneous implantations, JoMa1-ALK expressing cells rapidly induced tumor formation, and mice engrafted with JoMa1-ALK-F1174L cells developed tumors in AG much faster than JoMa1-ALK-R1275Q and JoMa1-ALK-wt bearing mice (p<0.02), while no tumor was found in mice implanted with JoMa1 or JoMa1-Migr control cells (Figure 3A). Exogenous ALK expression in orthotopic tumors was confirmed by RT-PCR, WB and IHC, while murine Alk was undetected (Figure 3B, and Supplementary Figure 3). Ki67 labeling revealed an enhanced proliferation index in JoMa1-ALK-F1174L-derived tumors (48%) compared to JoMa1-ALK-R1275Q- (40%), and JoMa1-ALK-wt-derived tumors (38%) (Figure 3b), suggesting that the accelerated tumor growth of JoMa1-ALK-F1174L tumors may partly result from their increased proliferation capacity.

Subcutaneous and orthotopic JoMa1-ALK-derived tumors correspond to highly malignant undifferentiated tumors (Figure 2E, and 3B), Phox2b^−^/Th^−^, but strongly expressing CD44, nestin, and weakly Ncam1 (Figure 3C). Tumors histologically close to NB, such as Ewing tumors, melanoma, or lymphoma were excluded, as their specific markers (CD99, Tyr, and Ptprc (CD45), respectively) were not detected (Figure 3C). Hence, JoMa1-ALK-wt, -F1174L, -R1275Q cells, similarly to MONC-1-ALK-F1174L cells, gave rise to undifferentiated NC cell-derived tumors.

Activation of ALK downstream signaling pathways analyzed in orthotopic tumors revealed phosphorylation of AKT, ERK and Stat3 in JoMa-ALK-F1174L tumors, while JoMa1-ALK-R1275Q tumors preferentially activated PI3K/AKT and Stat3 pathways, and JoMa1-ALK-wt tumors strongly activated PI3K/AKT and ERK pathways, while only weakly the Stat3 pathway (Supplementary Figure 4).

To further characterize JoMa1-ALK orthotopic tumors, their global gene expression profiles were analyzed using Affymetrix murine 430.2 arrays, and compared to transcriptomes of murine NB derived from TH-NMYC, ALKF^1174L^/DBHHiCre, and TH-NMYC/ALKF^1174L^/DBHHiCre transgenic models and adrenal glands previously analyzed by Heukamp *et al*. [23], as well as to transcriptome of JoMa1 cells and cell lines established from JoMa1-NMYC-derived tumors (mTu3-6) described by Schulte *et al*. [22], using the same Affymetrix arrays. Clustering results revealed that the three groups of JoMa1-ALK (F1174L, R1275Q, and wt) orthotopic tumors clustered together and close to JoMa1 and JoMa1-NMYC derived tumors cell lines, as well as to adrenal glands, while NB derived from murine transgenic models were much more distant (Supplementary Figure 5). This confirms that our orthotopic JoMa1-ALK-derived tumors were significantly different from murine NB models. This also indicates that variations found between JoMa1-ALK tumors and NB transgenic models did not result from differences between experimental procedures, as all JoMa1 samples (ours and those of Schulte *et al*.) clustered together (Supplementary Figure 5). Analyses of differentially regulated genes (FC>3 and p<0.05) revealed 1634 upregulated, and 1493 downregulated genes in orthotopic JoMa1-ALK tumors compared to transgenic NB tumors. Interestingly, various NCSC or SC associated markers, such as Myc, Sox2, Sox9, Notch1, Notch2, Snai2, Bmp1/2, Twist1/2, Hmga2, nestin, and vimentin were identified among the upregulated genes (Table 1), while sympathoadrenal lineage markers (Phox2a, Phox2b, Gata2, Gata3, Th, and Dbh), neuroendocrine markers (Chromogranin A, synaptophysin), neuronal markers (Neurofilament, Ncam1, and Uchl1) and Mycn were present in the downregulated genes (Table 1, and Figure 3D). These results confirm that in our NCPC model, ALK-wt or activating ALK variants drive the formation of highly undifferentiated NC cell-derived tumors, and may even impair NB tumor differentiation.

**Table 1:** Selected neural crest and stem cells associated markers upregulated (left), and sympathoadrenal lineage, neuronal, and neuroendocrine markers downregulated (right) in JoMa1-ALK-derived tumors compared to transgenic NB models

Genes upregulated in JoMa1-ALK-derived tumors			Genes downregulated in JoMa1-ALK-derived tumors		
Gene Symbol	Gene Name	Fold Change	Gene Symbol	Gene Name	Fold Change
Hmga2	high mobility group AT-hook 2	2720.3	Meis1	Meis homeobox 1	-3.0
Mmp9	matrix metallopeptidase 9	158.4	Ncam1	neural cell adhesion molecule 1	-5.6
Myc	myelocytomatosis oncogene	151.1	Uchl1	ubiquitin carboxy-terminal hydrolase L1	-10.1
Snai2	snail homolog 2 (Drosophila)	59.5	Nefl	neurofilament, light polypeptide	-11.9
Gja1	gap junction protein, alpha 1	58.9	Mycn	v-myc myelocytomatosis viral related oncogene, neuroblastoma derived (avian)	-16.0
Twist2	twist homolog 2 (Drosophila)	52.4	Gata2	GATA binding protein 2	-22.7
Twist1	twist homolog 1 (Drosophila)	41.5	Syp	synaptophysin	-30.4
Nes	nestin	39.4	Ret	ret proto-oncogene	-43.8
Junb	Jun-B oncogene	34.0	Alk	anaplastic lymphoma kinase	-72.7
Bmp2	bone morphogenetic protein 2	29.4	Mdk	midkine	-98.3
Wnt7b	wingless-related MMTV integration site 7B	28.2	Nefh	neurofilament, heavy polypeptide	-114.0
Pax3	paired box gene 3	27.6	Gata3	GATA binding protein 3	-188.9
Pdgfa	platelet derived growth factor, alpha	25.3	Dbh	dopamine beta hydroxylase	-216.8
Hoxb2	homeobox B2	25.0	Phox2a	paired-like homeobox 2a	-230.1
Klf4	Kruppel-like factor 4 (gut)	24.5	Phox2b	paired-like homeobox 2b	-292.5
Cd44	CD44 antigen	22.3	Chga	chromogranin A	-376.7
Met	met proto-oncogene	21.2	Nefm	neurofilament, medium polypeptide	-600.7
Cd34	CD34 antigen	19.0	Th	tyrosine hydroxylase	-1221.0
Sox9	Gene Name	18.9	Chgb	chromogranin B	-1301.9
Cebpb	CCAAT/enhancer binding protein (C/EBP), beta	15.9	Ddc	dopa decarboxylase	-3205.6
Bmp1	bone morphogenetic protein 1	12.6	Bex1	brain expressed gene 1	-4031.5
Fgfr1	fibroblast growth factor receptor 1	12.6			
Notch2	Notch gene homolog 2 (Drosophila)	11.5			
Sox2	SRY-box containing gene 2	8.7			
Vim	vimentin	3.5			
Hif1a	hypoxia inducible factor 1, alpha subunit	2.9			
Notch1	Notch gene homolog 1 (Drosophila)	2.7			
Pdgfb	platelet derived growth factor, B polypeptide	2.4			

**Figure 4:**
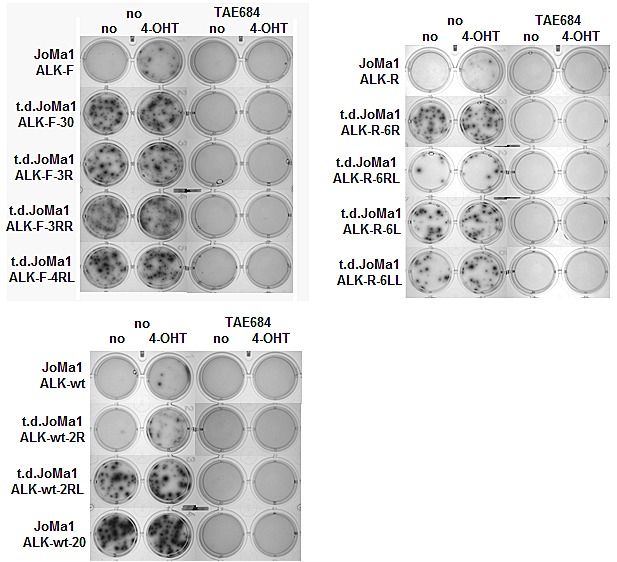
Tumor-derived cell lines become independent of Myc-ER^T^ activity, but remain dependent of ALK activity Clonogenic assays in methylcellulose with parental and t.d JoMa1-ALK cells incubated for 2 weeks in absence or presence of 4-OHT (200 nM), and/or TAE684 (1uM). One representative methylcellulose assay is shown.

### Tumor-derived JoMa1-ALK cells display an enhanced oncogenic potential compared to their parental cells

To further analyze tumorigenic properties of orthotopic tumor cells, cell lines were established upon tumors dissociation, which became independent of Myc-ER^T^ activity for long term passages *in vitro*. The clonogenic properties of t.d.JoMa1-ALK cell lines were then analyzed by semi-solid clonogenic assays in methylcellulose. T.d.JoMa1-ALK cell lines were able to produce numerous and large colonies in absence of 4-OHT, and addition of 4-OHT did not significantly increase colony number, or colony size, confirming their independency to Myc-ER^T^ activity, in contrast to parental cells, which were only able to form few macroscopic colonies in presence of 4-OHT (Figure 4). Furthermore, TAE684-mediated ALK inhibition completely abolished the clonogenic potential of t.d-JoMa1-ALK cell lines even in presence of 4-OHT (Figure 4), indicating that t.d-JoMa1-ALK cells display an enhanced oncogenic potential compared to their parental cells, but remained fully dependent on ALK activity.

### ALK mediates Myc upregulation in JoMa1-ALK tumors and tumor-derived cell lines, and both oncogenes cooperate in the oncogenic process

**Figure 5:**
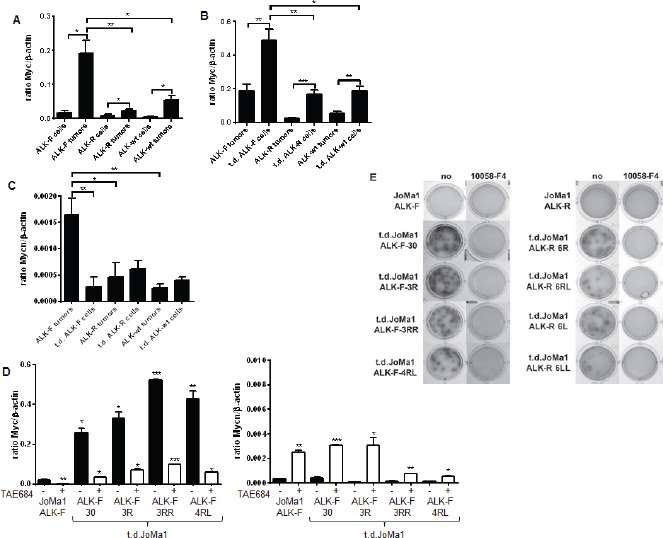
Myc expression is strongly enhanced in orthotopic tumors and in tumor-derived cell lines, and plays a strong oncogenic role *in vitro* A. Myc mRNA expression levels measured by semi-quantitative real-time qPCR in parental JoMa1 cells and in orthotopic tumors. Five representative samples were analyzed for JoMa1-ALK-F, and JoMa1-ALK-R tumors; 4 for JoMa1-ALK-wt tumors, t.d.JoMa1-ALK-F, and t.d.JoMa1-ALK-R cell lines; and 3 for t.d.JoMa1-ALK-wt cell line. To note: the exogenous Myc-ER^T^ is from human origin and was not detected by the murin Myc specific primers used. Mean ratio between Myc and β-actin as measured in three independent experiments are indicated (*=p<0.05, and **=p<0.005). B. Myc mRNA expression levels in orthotopic tumors, and in t.d.JoMa1 cell lines measured as in A (*=p<0.02, ***=p<0.005, and ***=p<0.0005). C. Mycn mRNA expression levels measured as in A. D. Myc and Mycn mRNA expression levels in ALK-F parental and t.d. cell lines treated or not with 1 μM TEA684 for 24h, and measured as in A (see different scales)(*=p<0.05, **=p<0.002). E. Pictures of one representative clonogenic assay in methylcellulose are shown. Cells were incubated for 2 weeks in absence or presence of 80uM Myc inhibitor (10058-F4).

Myc was found to be overexpressed in JoMa1-derived tumors compared to NB transgenic models, while Mycn was downregulated (Table 1). To validate these data, Myc and Mycn mRNA expression levels were measured in JoMa1-ALK tumors and in their respective parental or tumor-derived cell lines. Myc was upregulated in transduced JoMa1-ALK-F1174L and -ALK-R1275Q cells relative to JoMa1-Migr control cells (Supplementary Figure 6A). In addition, Myc mRNA expression level was increased in orthotopic and subcutaneous tumors compared to their respective parental JoMa1-ALK cells (Figure 5A, and Supplementary Figure 6B). Moreover, Myc was also significantly upregulated in t.d.cell lines as compared to their tumors of origin (Figure 5B). Furthermore, JoMa1-ALK-F1174L tumors, or t.d.JoMa1-ALK-F1174L cells, expressed significantly higher amounts of Myc mRNA compared to JoMa1-ALK-R1275Q- and JoMa1-ALK-wt-derived tumors, or -associated t.d.cell lines, respectively (Figure 5A, 5B, and Supplementary Figure 6B). Finally, qPCR analyses confirmed the particularly weak Mycn mRNA expression levels in JoMa1-ALK orthotopic tumors and t.d.cell lines relative to Myc levels (Figure 5C and, see the different scales). However, Mycn mRNA expression was superior in JoMa1-ALK-F1174L tumors relative to JoMa1-ALK-R and JoMa1-ALK-wt tumors (Figure 5C). Altogether, these results suggest that Myc, rather than Mycn, may have an essential role in the tumorigenesis of ALK-expressing JoMa1 cells *in vivo*.

To further investigate whether Myc upregulation observed in t.d.cells was dependent on ALK activity, tumor-derived and parental JoMa1 cells were treated with the ALK inhibitor TAE684 for 24h. Myc mRNA expression was strongly downregulated upon TAE684 treatment (Figure 5D, and data not shown for t.d.JoMa1-ALK-R and -ALK-wt cells). Interestingly, Mycn mRNA expression levels were inversely correlated to that of Myc, as Mycn was strongly upregulated in presence of TAE684, even if the Mycn/β-actin ratio remained extremely low compared to that of Myc (Figure 5D).

To analyze whether Myc upregulation participated to the increased tumorigenicity of t.d.JoMa1 cell lines, we measured their clonogenic capacity in presence or absence of the Myc inhibitor 10058-F4. Interestingly, *in vitro* tumorigenic capacities of t.d.JoMa1-ALK expressing cells were completely abolished by treatment with 10058-F4 (Figure 5E, and data not shown for t.d.JoMa1-ALK-wt cells). Overall these results suggest that Myc cooperate with ALK-F1174L, as well as with ALK-R1275Q and ALK-wt, in enhancing the clonogenic capacities of tumor-derived cells *in vitro* and possibly tumor growth *in vivo*.

## DISCUSSION

The role of ALK-wt, ALK-F1174L and ALK-R1245Q in NB tumor initiation was investigated in two NCPC models, MONC-1 and JoMa1. We have demonstrated by orthotopic implantations in mice that parental MONC-1 cells produced diverse tumor types, such as undifferentiated NB, osteo-chondrosarcoma, and undifferentiated tumors, recapitulating the pluripotency of MONC-1 NCPC. Interestingly, ALK-F1174L expression in MONC-1 cells not only strongly accelerated tumor growth, but also affected their differentiation capacity. Indeed, MONC-1-ALK-F1174L cells only developed highly malignant undifferentiated tumors expressing high levels of the neural stem/progenitor cell marker nestin. Similarly, we provide the first demonstration that even in absence of exogenous Myc-ER^T^ activity, ALK-wt, ALK-F1174L, or ALK-R1275Q expression in NCPC JoMa1 are sufficient to initiate highly aggressive, undifferentiated tumor formation, although not to drive NB progression. We have shown that MONC-1-ALK-F1174L- and JoMa1-ALK-derived tumors were nestin^+^ and Phox2b^−^. Interestingly, hyperplastic lesions observed in early post-natal sympathetic ganglia derived from TH-MYCN NB mice models contained nestin^+^/Phox2b^−^ cells. These cells were proposed to “represent a population of malignant sympathetic neural crest or early progenitor cells that give rise to Phox2b positive tumor cells comprising the bulk of NB tumors” [31, 32]. Thus, ALK expressing MONC-1- and JoMa1-derived tumors may correspond to such malignant NCPC populations.

Comparison of gene expression profiles between orthotopic JoMa1-ALK-derived tumors and NB transgenic mouse models of Heukamp *et al*. [23] revealed a strong upregulation of SC- or NCSC-associated markers, and a significant downregulation of differentiation markers related to neuronal or sympathoadrenal lineage in JoMa1-ALK-derived tumors. Thus, in our NCPC models, ALK expression in MONC-1 or JoMa1 cells appeared to maintain tumor cells in an undifferentiated state. The high Myc/Mycn ratio detected in our orthotopic tumors may partly explain their stem-like phenotype, as Myc is one of the few genes needed to induce pluripotent stem cell reprogramming [33]. Although produced in the same JoMa1 model, our results differ from Schulte *et al*. report, which show only a very weak tumorigenic and NB-inducing potential of JoMa1-ALK-F1174L cells after subcutaneous injection [22]. The selection of clones in Schulte study and variations in ALK-F1174L expression levels in JoMa1 cells are elements that could explain the discrepancies between both studies.

Understanding of mechanisms responsible for ALK-mediated inhibition of NCPC differentiation will require further investigations. NB tumors expressing activated ALK derived from *MYCN*/KI *ALK* mice displayed a more differentiated phenotype as compared to *MYCN* tumors derived from TH-MYCN mice [26]. ALK was also shown to induce differentiation of PC12 cells by inducing neurite outgrowth [34]. However, membrane attachment of ALK-tyrosine kinase domain was shown to be crucial for the control of neurite outgrowth and proliferation arrest, while cytoplasmic localization promoted cell proliferation [34]. Thus, ALK cellular localization may play a role in the switch between proliferation and differentiation. It should be noted that ALK localization in MONC-1- and JoMa1-ALK tumors was mostly cytoplasmic. Interestingly, ALK expression was recently shown to be associated with less differentiated neuroblastic tumors, as the frequency of ALK positivity in NB was significantly higher than in ganglioneuroblastoma, and in ganglioneuroma [35, 36].

Observations that frequent ALK-wt overexpression in NB primary tumors was associated with poor clinical outcome, similarly to activating ALK mutations [10, 17, 18, 37], suggested that ALK-wt overexpression may be involved in NB oncogenesis and progression. We demonstrate here for the first time that ALK-wt expression in NCPC JoMa1 can drive malignant tumor formation in nude mice. Until this study, ALK-wt transforming capacity could not be demonstrated neither in clonogenic assays nor upon subcutaneous implantations [13, 14, 38, 39]. Also, ALK-wt expression was unable to cooperate with MYCN in inducing NB formation in a zebrafish NB model [25]. These negative results may be explained by ALK-wt protein expression levels, which probably did not reach the critical threshold necessary for ALK-wt oncogenic autoactivation [40], while here we were able to detect a weak constitutive ALK phosphorylation in transduced JoMa1-ALK-wt cells.

The capacity of activating ALK mutations or ALK-wt overexpression to initiate tumor formation and progression may be dependent on their expression levels, as discussed above, as well as on their temporal expression during sympathetic neuronal development and differentiation. The production of a Tamoxifen-dependent Cre model allowing ALK activation at different development time points, as suggested [23], could help to determine whether a specific development period exists for NB tumor initiation and progression. Here, ALK was expressed in NCPC, which represent a very early stage of NC cell differentiation, as compared to ALK expression driven by Th or Dbh promoters in NB transgenic models. Indeed, Th and Dbh are expressed later in more differentiated cells of the sympathoadrenal lineage during noradrenergic induction of sympathetic neurons [1, 4]. The *bona fide* model to study the exact role of *ALK* mutations in NB tumor initiation and progression is the generation of ALK KI mutants, as recently described by Janoueix-Lerosey *et al*. [26]. In this study, the KI of the murine equivalents (ALK-F1178L and R1279Q in mice) of the two most frequent ALK activating mutations in patient were generated. *ALK* KI mice displayed in an enlargement in sympathetic ganglion resulting from enhanced proliferation of sympathetic neuroblasts [26]. However, expression of activating mutants did not generate tumors in mice in absence of MYCN expression, confirming that activated Alk was not sufficient to drive neuroblastic tumors [26].

So, our NCPC JoMa1 model revealed the strong tumor-initiating capacity of ALK-wt and activating mutants, in contrast to NB murine models. Indeed, in zebrafish and murine NB transgenic models, ALK-F1174L expression driven by Dbh or Th promoter, as well as activated *ALK* KI mutants, did not allowed to confer a fully transformed phenotype to NC derived cells, and thus to induce tumor formation, while co-expression of MYCN was required for NB development [24, 25, 26]. Also, ALK-F1174L required additional hits to drive NB development in the conditional actin-ALKF^1174L^;DBH-HiCre transgenic mouse model of Heukamp *et al*., as NB tumors occurred only in presence of additional genetic alterations [23]. Thus, ALK was not sufficient to drive NB formation both in NB murine models, and in our NCPC model.

We also confirmed the previously described remarkable tumorigenic potential of ALK-F1174L mutation [10, 13, 20-22, 38] in our NCPC MONC-1 and JoMa1 models. This can be in part be explained by the higher proliferation index of JoMa1-ALK-F1174L tumors. The enhanced tumorigenic potential of ALK-F1174L in the JoMa1 model may also be explained by the stronger upregulation of Myc in JoMa1-ALK-F1174L tumors compared to JoMa1-ALK-R1275Q and JoMa1-ALK-wt tumors. Increased Myc expression was also observed in all JoMa1-ALK tumors and tumors-derived cell lines, when compared to parental JoMa1-ALK cells. This may result from the *in vivo* selection of cells displaying enhanced survival and proliferation capacities. We demonstrated that Myc upregulation was predominantly dependent on ALK activity, as treatments of tumor-derived JoMa1-ALK-expressing cells with TAE684 induced strong downregulation of Myc mRNA. In addition, expression of activating ALK mutants in JoMa1 transduced cells upregulated Myc compared to control JoMa1-Migr cells. Upregulation of Myc was previously described in NMP/ALK-positive compared to NMP/ALK-negative lymphoma [41], suggesting that Myc may be an important downstream target of ALK signaling. The preferential ALK-mediated induction of Myc over Mycn, observed in our NCPC model as opposed to neuronal and NB cells [21, 24], may result from ALK expression in different developmental stages of NC-derived cells, which may express distinct stage-determining factors. In NB, Myc expression was shown to predominate over MYCN when both genes are expressed in absence of *MYCN* amplification or forced overexpression [42]. Conversely, Myc seems to be repressed by MYCN in *MYCN*-amplified NB, indicating regulatory interaction between Myc and MYCN expression [42]. Indeed, we observed that Myc mRNA downregulation, induced by ALK inhibitor treatment in tumor-derived JoMa1-ALK cells, was concomitant with Mycn upregulation. Thus, ALK-wt and activating mutants may not only upregulate MYCN mRNA expression, as shown in neuronal and NB cells [21], and cooperate with MYCN in NB tumorigenesis [21, 23-25], but they may also upregulate and cooperate with Myc, as observed in murine NCPC. Interestingly, the tumorigenic potential of t.d.-JoMa1-ALK expressing cells was strongly dependent on both ALK and Myc activities, as ALK or Myc inhibitor completely impaired their *in vitro* clonogenic potentials.

Therefore, our NCPC models allowed us to demonstrate the role of ALK-wt and the most frequent activating ALK mutations in NCPC in tumor initiation and in differentiation control. These models may also be valuable in the further identification of ALK-mediated mechanisms in oncogenesis.

## MATERIALS AND METHODS

### Culture of MONC-1 and JoMa1 cells

MONC-1 and JoMa1 cells were maintained in an undifferentiated state using the NC culture medium (NCC-medium [29]), supporting NCPC proliferation, composed of Dulbecco's Modified Eagle Medium (DMEM) F-12 (Invitrogen), 1% N2-Supplement (Invitrogen), 2% B27-Supplement (Invitrogen), 10 ng/ml EGF (R&D), 4 ng/ml FGFβ (PeproTech), 1% Penicillin-Streptomycin (Invitrogen), and 10% Chicken-Embryo-Extract [43]. Cells were cultivated in fibronectine coated dishes (1mg/ml, Sigma-Aldrich). For Myc-ER^T^ activation, 200 nM 4-OHT (Sigma-Aldrich) was added to NCC-medium.

### Retroviral infections

ALK cDNA *XhoI-EcoRI* fragments, isolated from ALK-wt-, ALK-F1174L-, and ALK-R1275Q-pcDNA3 constructs [44] were introduced into *XhoI-EcoRI* sites of the biscistronic retroviral vector pMigr1 encoding for eGFP [45]. All ALK-expressing vectors were verified by DNA sequencing. Retroviral infection were performed as described previously [46], and GFP^+^ cells sorted by FACS Aria Cell Sorter (Becton Dickinson). After expansion, transduced cells were frozen, and *in vitro* or *in vivo* assay were performed with only early cell passages.

### RNA preparation and RT-PCR

Total RNA from cell lines and tumors were extracted using RNeasy or miRNeasy Mini kits (Qiagen), respectively. CDNA were prepared as described earlier [47]. PCR using GoTaq Hot Start Kit (Promega) were performed with following primers: Ngfr-for 5'-GAATGCGAGGAGATCCCTGG-3', Ngrf-rev 5'-GGAGCAATAGACAGGAATGAGG-3', Snai1-for 5'-CACCCATACAGGTGAGAAGC-3', Snai1-rev 5'-TGTCCTGGATGACAGAACCA-3', Nefh-for 5'-GCAGCCAAAGTGAACACAGA-3, Nefh-rev 5'-CTGAATAGCGTCCTGGTAGG-3', Mash1-for 5'-TTGAACTCTATGGCGGGTTC-3', Mash1-rev 5'-GCCATCCTGCTTCCAAAGTC-3', human ALK-for 5'-TGTTGCCTCTCCTCGATGTG-3', human ALK-rev TGTCTTCTCCGCTAATGGTG-3', murine Alk-for 5'-TGCCAGAAGTGTGTTCAGAAC-3', murine Alk-rev 5'-CCCTTCCATGAAGGCTTCAG-3'. Primers for Snai2, Sox9, Sox10, Gfap, Gapdh were described [29]. Cycling reactions were 2min at 95°C followed by 35 cycles of 30s at 95°C, 30s at 60 to 65°C and 30s at 72°C, followed by 5min at 72°C.

### Real-Time qPCR

Expression levels of Myc, Mycn, and β-actin were assessed by semi-quantitative real-time qPCR as described [47] with the QuantiFast SYBR® green kit (Qiagen) and primers specific for murine Myc, Mycn, and β-actin (QuantiTect primer assay, Qiagen). Cycling conditions were 5min at 95°C, 40 cycles of 10s at 95°C, 20s at 60°C, and 1s at 72°C. Ratio of *Myc* or *Mycn* to *β*-actin gene expression was evaluated using the ΔCt method.

### Immunoblotting

JoMa1 cells were lysed in 10 mM Tris-Hcl (pH 7.4), 150 mM NaCl, 5 mM EDTA, 25 mM β-glycerophosphate, 10% glycerol, 1% NP40, 0.25% Na-deoxycholate, 20 mM NaF, 1 mM Na-pyrophosphate, 1 mM Na_3_VO_4_ and 1x protease inhibitor. Cell lysates were sonicated three times for 10 sec, and centrifugated at 13'000 rpm at 4°C during 30 min. Phosphoproteins were detected using polyclonal rabbit antibodies specific for phospho-ALK (pTyr1604, AssayBioTech), phospho-AKT (Cell Signaling), phospho-STAT3 (Cell Signaling) or phospho-ERK (Cell Signaling). Membranes were stripped 10min at 50°C in 2% SDS, 65.5 mM Tris-HCL pH 6.8 and 100 mM β-mercaptoethanol, and total protein expressions were detected using polyclonal rabbit antibodies specific for ALK (Invitrogen), AKT (Cell Signaling), STAT3 (Cell Signaling), or ERK (Cell Signaling).

### Methylcellulose clonogenic assay

JoMa1 cells (1.3*10^3^) were grown in 500 μl semi-solid medium [53% methylcellulose (Fluka, and prepared as in [48]), 17% FCS (Sigma), 30% DMEM (Gibco), and Penicilin-Streptomycin] in PolyHema (poly-2-hydroxyethyl-methacrylate, 16 mg/ml in EtOH, Sigma)-coated 24-well culture plates, to prevent cell adhesion. Tamoxifen (200nM), TAE684 (1μM) or 10058-F4 (80μM) were added twice a week in 100μl DMEM complemented with 10% FCS and Penicillin-Streptomycin. After 2 weeks, colonies were counted using an optic microscope, stained with a mix of 50% MTS/PMS (Promega) and 50% DMEM (Gibco) during 2 hours, and pictures were taken with AlphaImager®.

### *In vivo* studies

All animal experiments were carried out with female, athymic Swiss nude mice (BALB/C nu/nu), 4-6 weeks of age purchased from Charles River in accordance to the European Community guidelines (directive no. 86/609/CEE) as described [49, 50]. In subcutaneous model, 5x10^5^ cells were injected in both flanks of mice (3 per group). In orthotopic model, 10, or 7 mice per groups were injected in left AG with 10^5^ MONC-1 cells, or 1.5x10^5^ JoMa1 cells, respectively, resuspended in 10 μl of PBS. Abdominal incisions were closed with skin clips.

Tumor take and growth were followed up using calipers twice a week for subcutaneous injections or by ultrasound every 3, 7, or 14 days according to tumor progression. Subcutaneous or orthotopic tumor volumes were calculated using the formula (length×width^2^)/2, or 4/3×π×(depth×sagittal×transversal)/6 formula, respectively. Mice with tumor volumes greater than ~1000 mm^3^, or ~800 mm^3^ were sacrificed for subcutaneous, or orthotopic injections, respectively. Every tumor was split into pieces for paraffin-embedded tissue formation, RNA or protein extraction, or cell dissociation.

### Tumor dissociation and establishment of tumor-derived cell lines

Cells were dissociated from orthotopic tumors as described earlier [51] and propagated like parental cells. Tumor-derived cell purity was analyzed after 2 weeks by flow cytometry for GFP expression.

### Immunohistochemistry

All immuno-labeling were performed by the Lausanne Mouse Pathology Facility. Hematoxylin and eosin (H&E) staining was performed on all subcutaneous and orthotopic tumors. Immunohistochemistry (IHC) was performed using mouse monoclonal Ab anti-TH (MAB318, Millipore), rabbit polyclonal Ab anti-ALK (51-3900, Invitrogen), anti-Tyr (a gift from Bhushan Sarode, EPFL), anti-CD99 (Orb13719, Biorbyt), anti-Ncam1 (14255-1-AP, Proteintech), rat monoclonal Ab anti-CD44 (550538, BD Pharmingen), anti-Ptprc (CD45) (550539, BD Pharmingen), anti-nestin (MAB353, Millipore), anti-Ki67 (M7249, DAKO), anti-Phox2b (H-20, Santa Cruz Biotechnology).

Ki67 proliferation index was determined by analyzing 5 fields imaged at 40x magnification per tumor section in 4 different tumors from each group. Sections were imaged using a Zeiss Axio LabA1 microscope and Axiovision Rel. 4.5 imaging software.

### Microarray analyses

CDNA, prepared from total RNA of orthotopic tumors, were hybridized to the Affimetrix GeneChip® Murine 430.2 oligonucleotide Array according to manufacturer's instructions. Arrays were normalized by the GCRMA procedure using Brainarray annotations [52].

### Statistical analysis

Statistical significance of results was analyzed using t-test and one-way ANOVA analysis using GraphPad Prism 5.04 software (GraphPad Software, Inc.).

## SUPPLEMENTARY FIGURES


