# Supplementary material for: Wild-type ALK and activating ALK-R1275Q and ALK-F1174L mutations upregulate Myc and initiate tumor formation in murine neural crest progenitor cells
Source: Oncotarget. 2014 May 27;5(12):4452–66. doi: 10.18632/oncotarget.2036 (PMC4147337; doi:10.18632/oncotarget.2036)
Supplement: Supplementary file 1 [file oncotarget-05-4452-s001.pdf]

Wild-type ALK and activating ALK-R1275Q and ALK-F1174L mutations upregulate Myc and initiate tumor formation in murine neural crest progenitor cells – Montavan et al

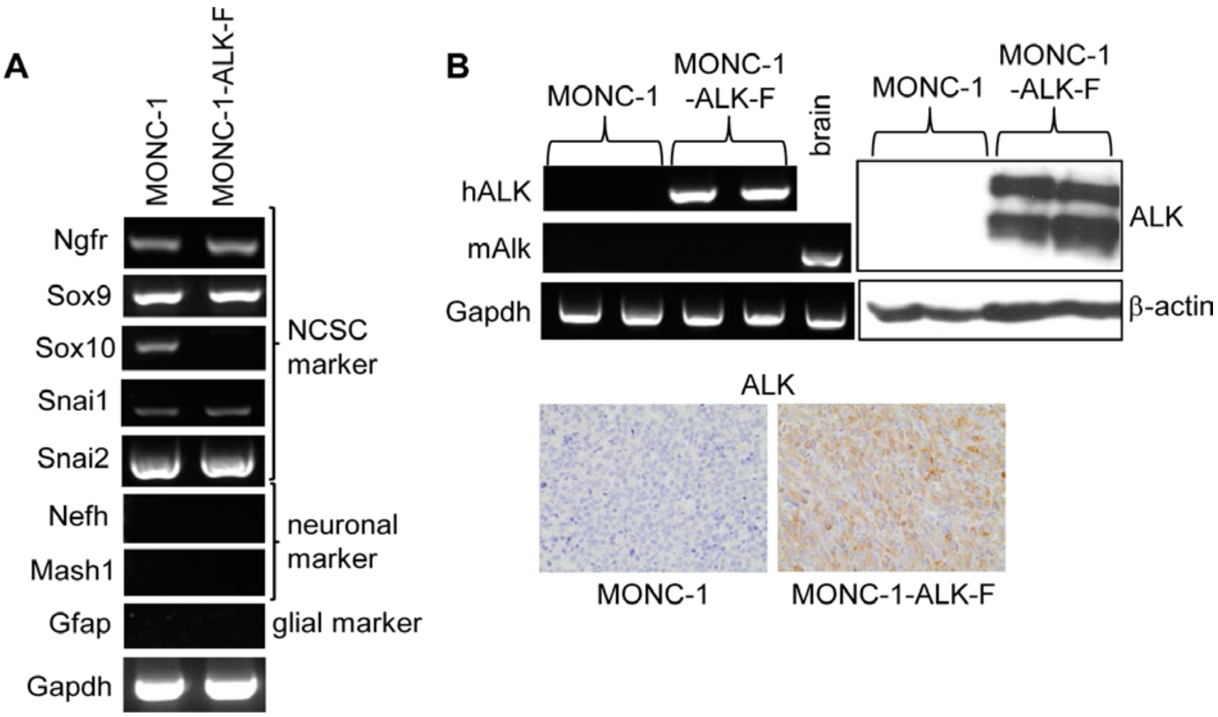

Supplementary Figure 1: A. Expression levels of various NCSC and differentiation markers were analyzed by RT-PCR in total RNA from MONC-1 parental and MONC-1-ALK-F1174L cells. Murine Gapdh was used as internal control B. ALK mRNA and protein expression in orthotopic tumors derived from MONC-1 and MONC-1-ALK-F1174L cells was assessed by RT-PCR and immunoblotting (upper panel, 2 representative tumors) and by IHC (lower panel, magnification 40x, 1 representative tumor). Gapdh and β-actin were used as loading control for RT-PCR and WB, respectively.

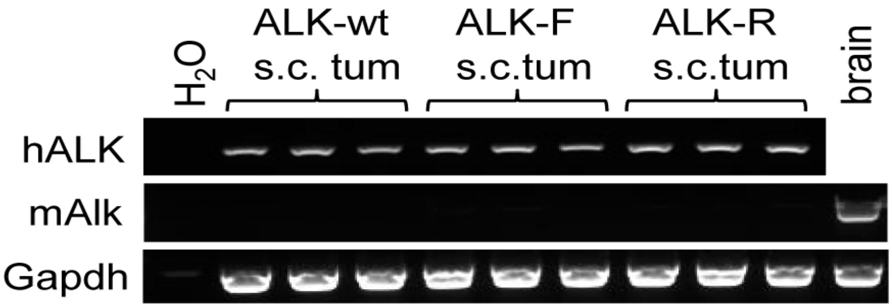

Supplementary Figure 2: RT-PCR analyses of human and murine ALK expression in subcutaneous tumors. Gapdh was used as internal control.

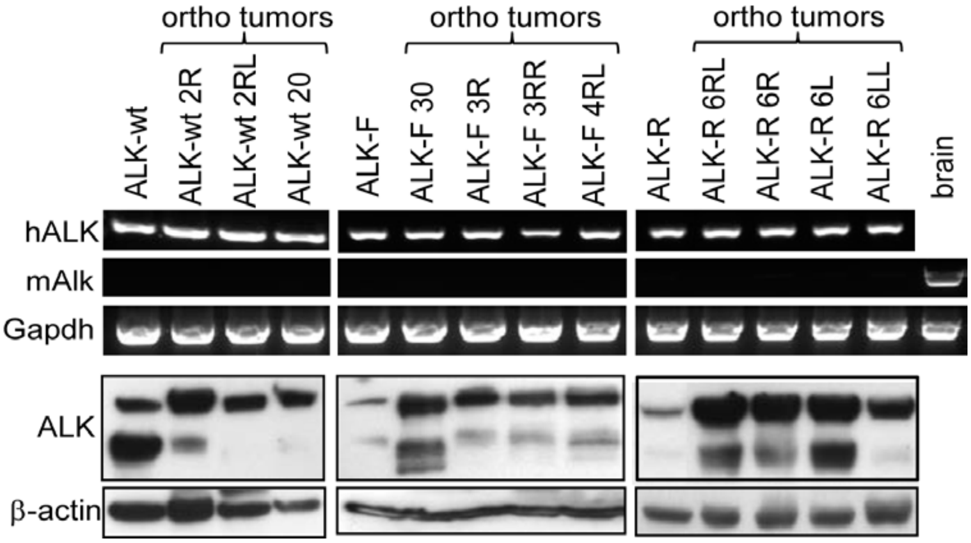

Supplementary Figure 3: RT-PCR (up) and immunoblotting (down) analyses of ALK expression in orthotopic tumors derived from JoMa1-ALK-F1174L, -ALK-R1275Q, and -ALK-wt cells, as well as parental cells. Gapdh and β-actin were used as loading control for RT-PCR and WB, respectively.

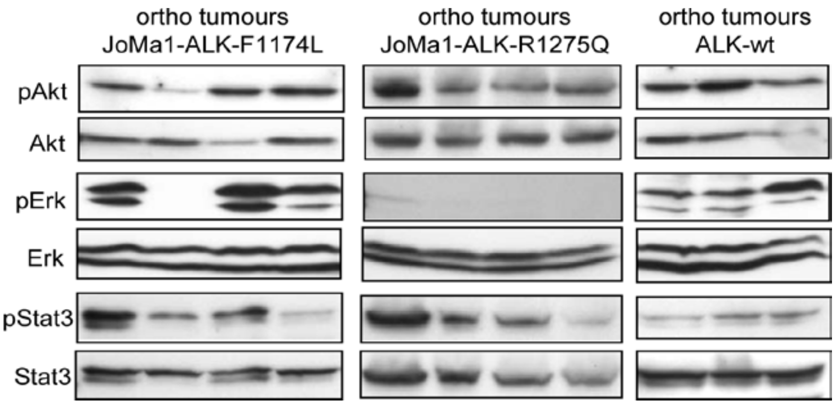

Supplementary Figure 4: Immunoblotting analyses of AKT, ERK, and Stat3 phosphorylation in 4 representative orthotopic tumors derived from either JoMa1-ALK-F1174L or ALK-R1275Q cells, and in 3 JoMa1-ALK-wt-derived orthotopic tumors. Total AKT, ERK, and Stat3 expression were used as loading control.

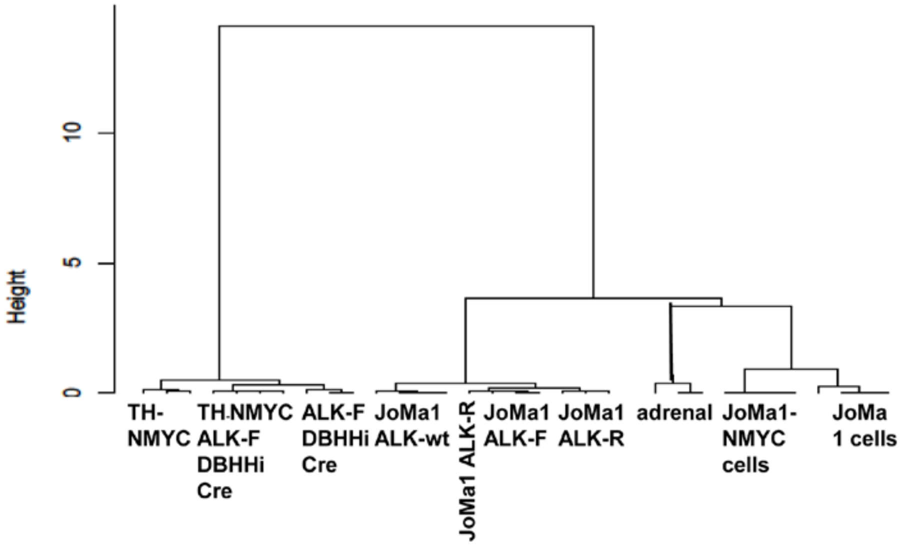

Supplementary Figure 5: Analysis of gene expression profiles of JoMa1-ALK expressing orthotopic tumors. Unsupervised hierarchical clustering of JoMa1-ALK-expressing tumors with published murine NB models and AG from Heukamp et al. [23], and published JoMa1 cell and cell lines established from JoMa1-NMYC tumors from Schulte et al. [22], performed using the top 5% genes with the highest IQR.

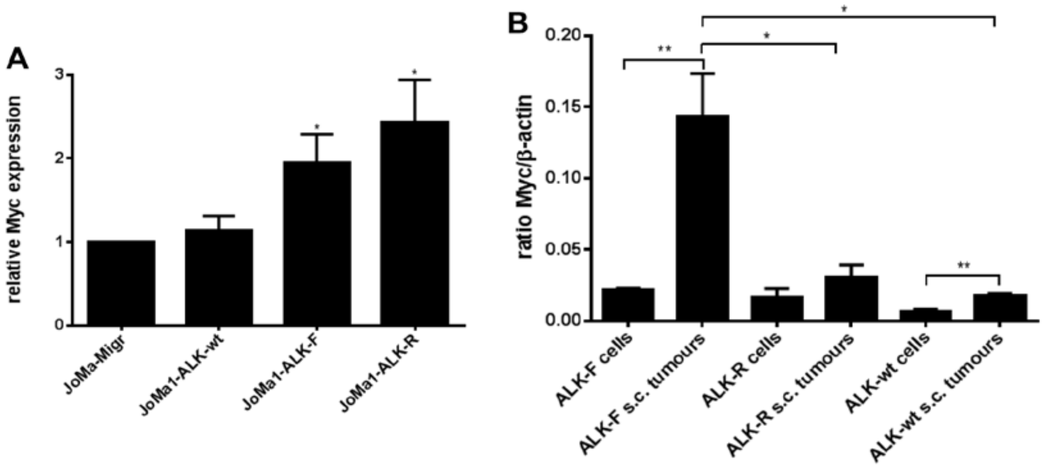

Supplementary Figure 6: A. Myc mRNA expression levels measured by semi-quantitative real-time qPCR in JoMa1 transduced cells. Mean ratio of Myc and β-actin of three independent experiments are indicated (*p< 0.0202). B. Myc mRNA expression levels measured by semi-quantitative real-time qPCR in parental JoMa1-ALK-expressing cells and in corresponding subcutaneous tumors. Three different tumors from JoMa1-ALK-F1174L, JoMa1-ALK-R1275Q, and JoMa1-ALK-wt groups were analyzed (*=p<0.03, and **=p<0.001).
